# Google DeepMind’s gemini AI versus ChatGPT: a comparative analysis in ophthalmology

**DOI:** 10.1038/s41433-024-02958-w

**Published:** 2024-02-14

**Authors:** Mouayad Masalkhi, Joshua Ong, Ethan Waisberg, Andrew G. Lee

**Affiliations:** 1 University College Dublin School of Medicine, Belfield, Dublin Ireland; 2 Department of Ophthalmology and Visual Sciences, University of Michigan Kellogg Eye Center, Ann Arbor, MI USA; 3 Department of Ophthalmology, University of Cambridge, Cambridge, UK; 4 Moorfields Eye Hospital, NHS Foundation Trust, London, UK; 5 Center for Space Medicine, Baylor College of Medicine, Houston, TX USA; 6 The Houston Methodist Research Institute, Houston Methodist Hospital, Houston, TX USA; 7 Departments of Ophthalmology, Neurology, and Neurosurgery, Weill Cornell Medicine, New York, NY USA; 8 Department of Ophthalmology, University of Texas Medical Branch, Galveston, TX USA; 9 University of Texas MD Anderson Cancer Center, Houston, TX USA; 10 Texas A&M College of Medicine, Bryan, TX USA; 11 Department of Ophthalmology, The University of Iowa Hospitals and Clinics, Iowa City, IA USA

**Keywords:** Eye manifestations, Risk factors

## Introduction

Google’s Gemini AI represents a significant leap in chatbot technology, showcasing advanced capabilities and innovative features. Central to Gemini’s design is its status as a “native multimodal” model, enabling it to process and learn from various data types, including text, audio, and video. Gemini’s technical capabilities is evident in its ability to analyse complex data sets, such as charts and images, which is a substantial advancement over the earlier Bard AI models [1]. This capability is particularly relevant for applications in medicine and ophthalmology, where data often comes in visual formats like medical images/scans. By analysing these images, Gemini could potentially be a useful tool to healthcare professionals in diagnosing and treating a wide range of conditions.

Moreover, Gemini’s potential in medicine extends beyond image analysis. Its advanced language processing abilities enable it to understand and interpret medical literature, patient histories, and research data, providing valuable insights for medical professionals. In ophthalmology, Gemini could assist in diagnosing eye conditions, analysing patient-reported symptoms, and even suggesting treatment plans based on the latest research and clinical guidelines. ChatGPT has previously attempted these tasks, however did not yet perform at suitable levels to be used clinically [2–7]. Large language models such as ChatGPT can make errors in understanding the context of information, or provide outdated information, which further complicates the usage of these technologies in a clinical context [8–11].

**Fig. 1:**
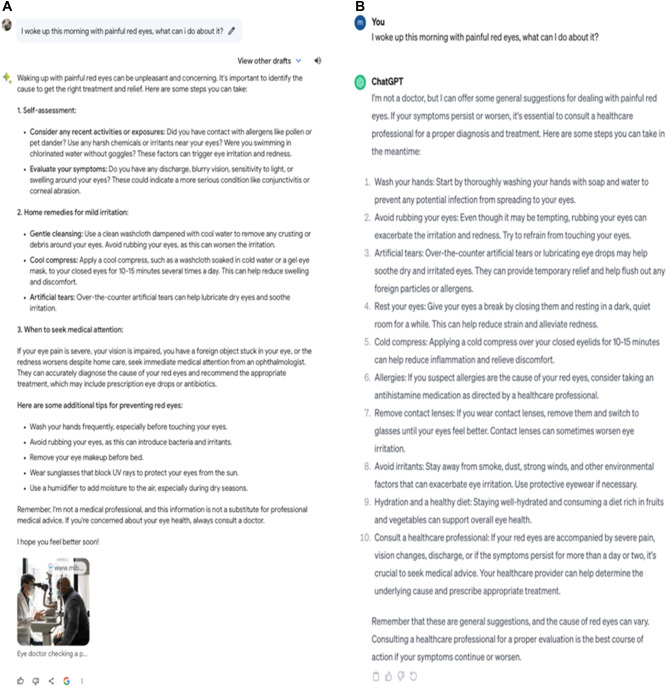
Output responses generated by Bard and ChatGPT. **A** Output generated by Bard from the prompt “I woke up this morning with painful red eyes, what can I do about it?”. **B** Output generated by ChatGPT 3.5.

We first decided to ask Bard to advise a patient of what to do when they complained of waking up with painful red eyes. Bard’s response was thorough and practical, providing a list of steps the patient could take, such as applying cool compress, using artificial tears, and avoiding eye rubbing, to relieve any on-going inflammation (Fig. 1A). ChatGPT similarly provided very similar, yet a longer more comprehensive list of practical guidance and steps that the patient could take to reduce their discomfort. Bard and ChatGPT’s responses were medically sound and in-line with current clinical guidelines.

Next, we asked Gemini about how often an individual should have an eye exam. Gemini AI suggested four age-based recommendations for eye exams, noting that individual needs may vary due to factors like eyeglass use, existing eye conditions, or family medical history. Similarly, ChatGPT had categories of ‘Children and teenagers’, ‘Adults’, and ‘Older adults’. Both Gemini AI and ChatGPT highlighted the importance of consulting with an eye specialist.

Next we prompted both of AI chatbots about a patient reporting “flashes of lights” in one eye, and if they should attend the emergency department. Both Bard and ChatGPT correctly recommended to attend the emergency department, particularly if this vision change occurred suddenly. Both chatbots also appropriately stated that this symptom could be a sign of a retinal tear or detachment, requiring urgent evaluation. These AI-generated outputs were both specific, and appropriate.

**Fig. 2:**
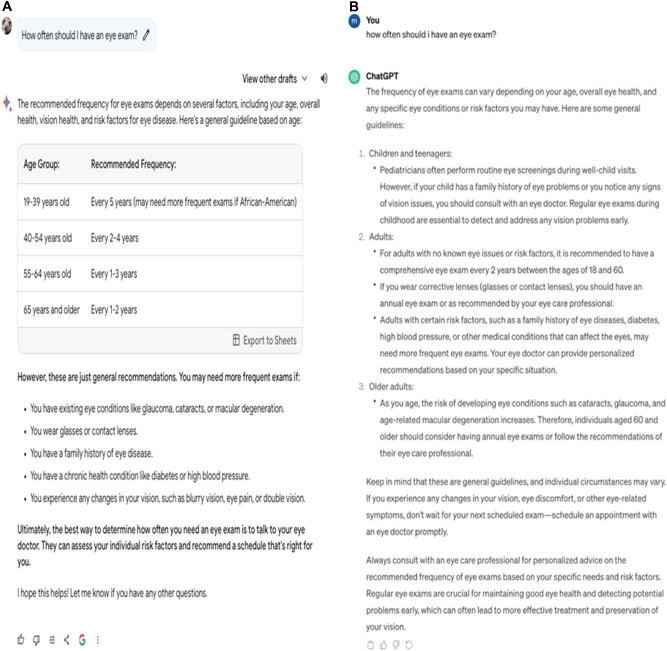
Output responses generated by Bard and ChatGPT. **A** Output generated by Bard from the prompt “**How often should I have an eye exam?”** (Left Panel). **B** Output generated by ChatGPT 3.5. (Right Panel).

**Fig. 3:**
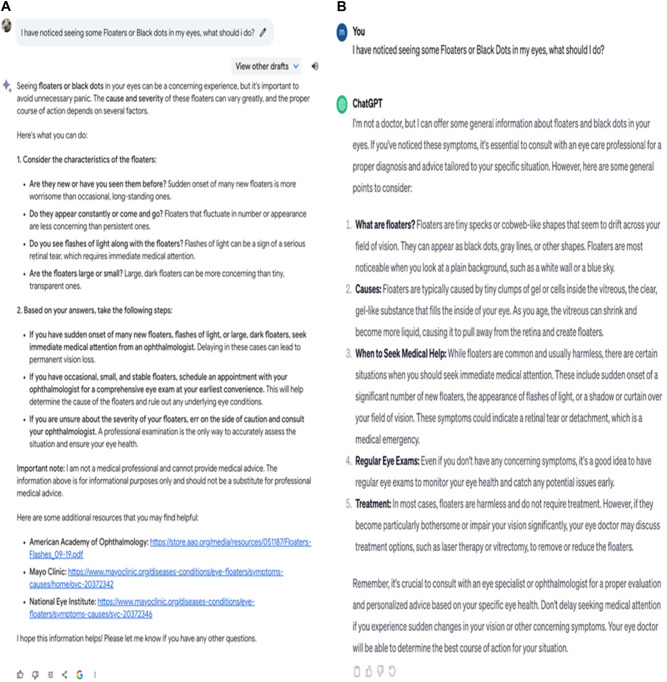
Output responses generated by Bard and ChatGPT. **A** Output generated from the prompt “I have noticed seeing some **Floaters or Black Dots in my eyes, what should I do?**” (Left Panel). **B** Generated by ChatGPT 3.5. (Right Panel).

Finally, we prompted both AI chatbots about what a patient should do if they started seeing floaters or black dots (see Fig. 2). There are several causes of floaters ranging from relatively benign (e.g. age-related) to more serious causes (e.g. retinal detachment). Bard accurately reported a few potential reasons and suggested a formal consultation with an eye care specialist if sudden blindness developed or if the patient started experiencing changes in floater size or light flashes, which correctly addresses potential risk Bard also provided practical tips to reduce discomfort due to floaters. ChatGPT’s response was similar to Bard and also correctly explained causes of floaters and when to seek urgent medical assistance. ChatGPT, unlike Bard, also provided information on floaters treatment. In addition, ChatGPT advised seeing an eye doctor if there were several floaters, light flashes or a seeing a curtain over the vision field (Fig. 3).

Finally, we wanted to test the image analysis capabilities of Gemini AI against GPT-4.

**Fig. 4:**
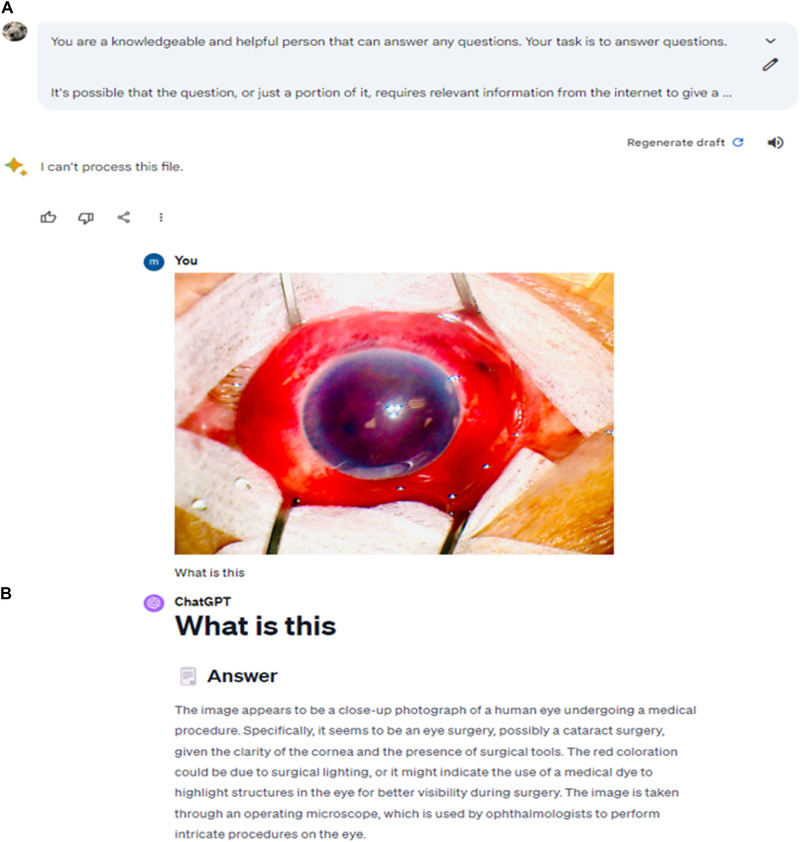
Output responses generated by Bard and ChatGPT. **A** Response by Gemini AI (Top Panel). **B** Response by GPT-4 (Bottom Panel). Reprinted without changes from “Cheers not tears: champagne corks and eye injury, 10.1136/bmj.p2520.

Gemini AI unfortunately could not process the file despite attempting a variety of prompts. On the other hand, GPT-4 correctly identified the image of a human eye and that the picture was taken using an operating microscope. However, GPT-4 failed to correctly describe the red coloration as hyphaema (Fig. 4).

## Conclusion

Overall, the new Gemini AI model represents a notable improvement in text-based output than predecessor models. The comparative analysis between Gemini AI and ChatGPT/GPT-4 reveals distinct attributes and capabilities of these advanced AI models. Gemini AI shows promise with unique strengths in areas such as language understanding. It emerges as a strong competitor to ChatGPT, suggesting a dynamic and evolving landscape in AI language models. Both models exhibit exceptional capabilities but differ in various aspects of language processing and response generation. The analysis underlines the fact that each AI model, including ChatGPT, GPT-4, Bard, and Gemini AI, possesses unique strengths and weaknesses, making them suitable for different applications and use cases. It is important to note that further advancements are necessary prior to being the use of AI chatbots in clinical settings [12, 13].

## References

[1] Waisberg E, Ong J, Masalkhi M, Zaman N, Sarker P, Lee AG et al. Google’s AI chatbot “Bard”: a side-by-side comparison with ChatGPT and its utilization in ophthalmology. Eye. (2023). 10.1038/s41433-023-02760-0. 10.1038/s41433-023-02760-0 PMC10920702 37770534

[2] Waisberg E, Ong J, Masalkhi M, Kamran SA, Zaman N, Sarker P (2023). GPT-4: a new era of artificial intelligence in medicine. Ir J Med Sci.

[3] Shemer A, Cohen M, Altarescu A, Atar-Vardi M, Hecht I, Dubinsky-Pertzov B et al. Diagnostic capabilities of ChatGPT in ophthalmology. Graefes Arch Clin Exp Ophthalmol. (2024). 10.1007/s00417-023-06363-z. 10.1007/s00417-023-06363-z 38183467

[4] Waisberg E, Ong J, Masalkhi M, Kamran SA, Zaman N, Sarker P (2023). GPT-4 and ophthalmology operative notes. Ann Biomed Eng.

[5] Mihalache A, Popovic MM, Muni RH (2023). Performance of an artificial intelligence chatbot in ophthalmic knowledge assessment. JAMA Ophthalmol.

[6] Waisberg E, Ong J, Masalkhi M, Lee AG. Large language model (LLM)-driven chatbots for neuro-ophthalmic medical education. Eye. 2023;25:1–3. 10.1038/s41433-023-02759-7 PMC10920622 37749374

[7] Antaki F, Touma S, Milad D, El-Khoury J, Duval R (2023). Evaluating the performance of ChatGPT in ophthalmology. Ophthalmol Sci.

[8] Kocoń J, Cichecki I, Kaszyca O, Kochanek M, Szydło D, Baran J (2023). ChatGPT: jack of all trades, master of none. Inf Fusion.

[9] Waisberg E, Ong J, Kamran SA, Masalkhi M, Zaman N, Sarker P (2023). Bridging artificial intelligence in medicine with generative pre-trained transformer (GPT) technology. J Med Artif Intell.

[10] Jeyaraman M, Ramasubramanian S, Balaji S, Jeyaraman N, Nallakumarasamy A, Sharma S (2023). ChatGPT in action: Harnessing artificial intelligence potential and addressing ethical challenges in medicine, education, and scientific research. World J Methodol.

[11] Waisberg E, Ong J, Masalkhi M, Zaman N, Kamran SA, Sarker P (2023). ChatGPT and medical education: a new frontier for emerging physicians. Can Med Ed J.

[12] Alser M, Waisberg, E. Concerns with the usage of ChatGPT in Academia and Medicine: A viewpoint. Am J Med Open. 100036 (2023). 10.1016/j.ajmo.2023.100036. 10.1016/j.ajmo.2023.100036 PMC11256230 39035060

[13] Waisberg E, Ong J, Zaman N, Kamran SA, Sarker P, Tavakkoli A (2023). GPT-4 for triaging ophthalmic symptoms. Eye.

